# The psoriasis-associated IL-17A induces and cooperates with IL-36 cytokines to control keratinocyte differentiation and function

**DOI:** 10.1038/s41598-017-15892-7

**Published:** 2017-11-15

**Authors:** Carolina M. Pfaff, Yvonne Marquardt, Katharina Fietkau, Jens M. Baron, Bernhard Lüscher

**Affiliations:** 1 Institute of Biochemistry and Molecular Biology, Medical School, RWTH Aachen University, 52074 Aachen, Germany; 2 Department of Dermatology and Allergology, Medical School, RWTH Aachen University, 52074 Aachen, Germany

## Abstract

Psoriasis is a T_H_17-driven inflammatory disease affecting a significant proportion of the world population. The molecular consequences of IL-17 signaling in the skin are only partially understood. Therefore, we evaluated the IL-17A effects on organotypic 3-dimensional skin models and observed that IL-17A interfered with keratinocyte differentiation. In agreement with this phenotype, IL-17A repressed the expression of many genes encoding structural proteins. Moreover, genes encoding anti-microbial peptides were induced, resulting in a strengthening of the chemical barrier. Finally, we observed enhanced expression of the three IL-36 cytokines IL-36α, β and γ. We found that IL-36γ was secreted from keratinocytes in an inactive form and that neutrophilic proteases, including elastase, were capable of activating this cytokine. Functionally and similar to IL-17A, truncated IL-36 cytokines interfered with keratinocyte differentiation in 3D models. The molecular analysis revealed strong cooperative effects of IL-17A and IL-36 cytokines in regulating target genes, which was dependent on the proteolytic activation of the latter. Together these findings suggest an amplification cycle that can be initiated by IL-17A, involving IL-36 cytokines and immune cell derived proteases and resulting in active IL-36 cytokines which synergize with IL-17A. This amplification cycle might be relevant for a persistent psoriatic phenotype.

## Introduction

Psoriasis, a chronic autoimmune disease of the skin, affects approximately 2% of the population. Psoriasis is associated with systemic inflammatory processes including inflammatory arthritis, inflammatory bowel disease, and metabolic syndrome^1,2^. A typical manifestation of the disease is the hyperproliferation of keratinocytes with premature differentiation. This results in incomplete cornification with retention of nuclei in the stratum corneum, referred to as parakeratosis. Functionally this correlates with increased infiltrations of immune cells due to dendritic cells, macrophages, neutrophils and different subpopulations of T cells. These cells modify the cytokine milieu and thus affect the behavior of keratinocytes resulting in altered differentiation^1–3^.

Psoriasis is a T cell-mediated inflammatory disease with a suspected autoimmune pathogenesis. Autoantigens that have been found associated with psoriasis include cathelcidin/LL-37 and ADAMTS-like protein 5^4,5^. These promote the production of interleukin (IL) 23 by dendritic cells. Subsequently IL-23 stimulates T_H_17 cells, among others, that produce IL-17. The IL-23 – IL-17 axis is considered as particularly important in psoriasis based on various lines of evidence, including *ex vivo* experiments, mouse models, and transcriptomics studies^6^.

Soon after its discovery as a proinflammatory cytokine, IL-17 was recognized to be involved in psoriasis^7^. For example, IL-17 was shown to cooperate with interferon γ to stimulate the production of proinflammatory cytokines, including IL-6 and IL-8, in keratinocytes^8^. This is consistent with the increased frequencies of T_H_17 and T_H_1 cells in psoriatic skin, while in contrast in AD skin T_H_2 cells are elevated^9,10^. Unlike in psoriasis, IL-17 has little influence on the development of atopic dermatitis (AD), another common inflammatory skin disease, which is mainly driven by T_H_2 cytokines such as IL-4, IL-13, IL-22 and IL-31^11,12^. Of note, the Asian AD phenotype combines features of both AD and psoriasis with increased T_H_17 polarization^13^. The central role of IL-17 in psoriasis is most vividly documented by the success of Secukinumab, a fully human monoclonal antibody, which selectively targets IL-17A. It is highly effective in the treatment of severe plaque psoriasis and psoriatic arthritis^14,15^.

Expression studies revealed that IL-17 controls genes that encode for proteins associated with differentiation, with anti-microbial defense and with immune cell recruitment and activation, including filaggrin, S100A7, DEFB4, CCL20, and IL-8 (CXCL8)^16,17^. Many of these genes were confirmed when gene expression of psoriatic skin lesions was compared with control samples^18–20^. Together, these and other studies provide mechanistic information for the differentiation defect of keratinocytes observed in psoriatic lesions. Moreover, the expression of chemokines and cytokines, such as CCL20 and IL-8, promote the recruitment of immune cells, including neutrophils, that can further aggravate the inflammatory progression of psoriatic lesions through crosstalk with keratinocytes^21^.

Members of the IL-36 family, which are IL-1 type cytokines, have also been implicated in psoriasis^22,23^. IL-36 cytokines are involved in the crosstalk between immune cells and keratinocytes and thus are potentially participating in controling the proinflammatory milieu in psoriatic skin^24–30^. Moreover, interaction of IL-17 and IL-36 has been postulated to promote inflammation^26,31^. Similar to IL-17, IL-36 also activates anti-microbial peptides^32,33^. Importantly and similar to other IL-1 family cytokines, the different IL-36 proteins need to be processed to become biologically active. This occurs by truncating the N-termini, removing a few amino acids in response to several different proteases^34–36^. Thus, in addition to IL-17, IL-36 cytokines are also thought to participate in the inflammatory progression of psoriasis.

We used 3D organotypic skin equivalents to address the role of IL-17A in keratinocyte differentiation. IL-17A deregulated genes encoding differentiation markers and anti-microbial peptides preventing efficient keratinocyte differentiation and interfering with bacterial growth, respectively. In addition, IL-17A stimulated IL-36 type cytokine expression. Similar to IL-17A, active forms of IL-36 interfered with keratinocyte differentiation. IL-36 secreted from keratinocytes was inactive but could be activated by proteases-derived from stimulated neutrophils. Active IL-36 cytokines cooperated strongly with IL-17A in stimulating gene transcription, suggesting that an IL-17–IL-36 feed forward axis promotes and amplifies the inflammatory processes associated with psoriasis.

## Results

### IL-17A disturbs keratinocyte differentiation in 3D organotypic skin equivalents

**Figure 1:**
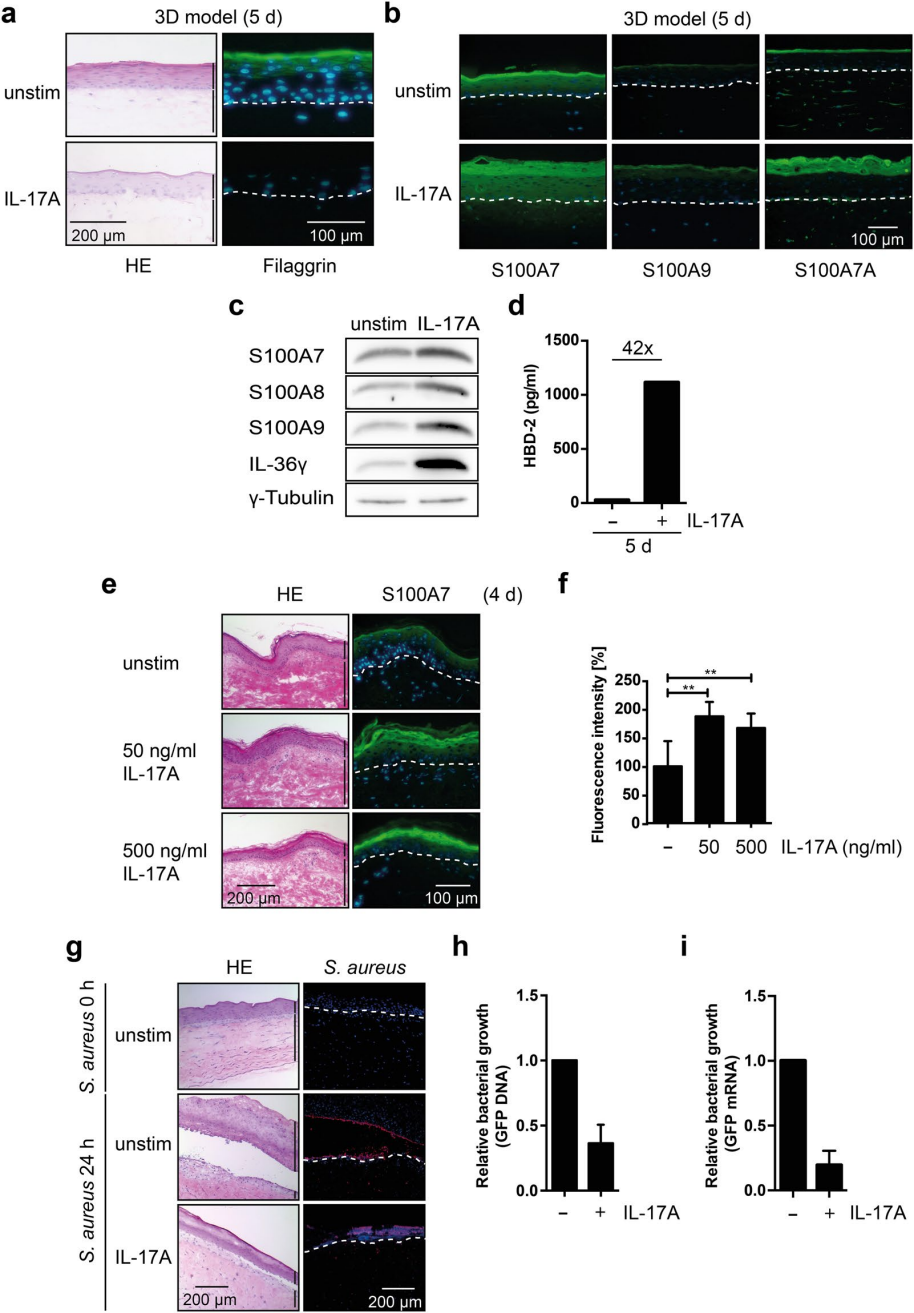
IL-17A interferes with keratinocyte differentiation. (**a**) NHEK organotypic 3D skin equivalents (3D models) were stimulated with or without IL-17A for 5 d. Histological sections were stained with H&E and for filaggrin (green), and the DNA was visualized with DAPI (blue). (**b**) Histological sections of 5 d NHEK 3D models stimulated with or without IL-17A were stained for the indicated AMPs (green), DNA is blue. (**c**) NHEK 3D models stimulated with or without IL-17A for 5 days were harvested and lysed in RIPA buffer. The indicated AMPs were analyzed on Western blots. The entire blots are displayed in Supplementary Fig. S10a. (**d**) The supernatants of NHEK 3D models stimulated for the indicated times with or without IL-17A were analyzed for hBD-2 protein expression in the culture supernatant by ELISA. (**e**) Human *ex vivo* skin explants were cultivated with and without 50 or 500 ng/ml rhIL-17A and harvested 4 days later. Histological sections were stained for S100A7 (green), DNA is blue. (**f**) Quantification of S100A7 fluorescence intensity of three sections of the *ex vivo* models shown in panel (e). **p < 0.01. (**g**) Normal NHEK 3D models were treated with or without IL-17A for 5 days as indicated. Subsequently, *S. aureus* stably expressing GFP were applied to the apical surface of the 3D model and incubated for 24 hours. The models were harvested and sections were stained with H&E. *S. aureus* was visualized using an antibody that recognizes an *S. aureus* peptidoglycan (red). The DNA was stained with DAPI (blue). (**h**) DNA was prepared and the integrated *GFP* transgene was quantified using PCR and relative bacterial growth calculated. Shown are mean values ± SD of five experiments. (**i**) GFP mRNA was measured using qRT-PCR. The relative bacterial growth was calculated, and mean values ± SD of two experiments measured in duplicates are displayed. Two bars on the right side of the H&E stainings indicate epidermis (upper bar) and dermis (lower bar) in the skin models. In the immunofluorescent stainings the basal layer is marked with a dotted line.

Because of the role of IL-17A in psoriasis and its effects on gene expression in primary normal human epidermal keratinocytes (NHEKs)^16,17^, we tested whether IL-17A affected the differentiation of keratinocytes in organotypic 3D skin equivalents (3D models) using keratinocytes and fibroblasts derived from skin biopsis of healthy individuals. We observed a strong inhibition of differentiation in response to IL-17A as visualized by hematoxylin & eosin (H&E) and filaggrin staining at day 5 (Fig. 1a). The consequences of IL-17A treatment in the 3D models were evaluated by comparing gene expression patterns. Genes encoding differentiation-associated proteins, chemokines and cytokines, and anti-microbial peptides (AMPs) were deregulated (Supplementary Fig. S1a), with some overlap to genes regulated by IL-17 in keratinocytes monolayer cultures^16^. Gene ontology (GO) analysis identified groups of IL-17A responsive genes associated with keratinocyte differentiation, skin barrier formation, and the response to bacteria (Supplementary Fig. S1b).

The expression of genes encoding AMPs and IL-36 family members were strongly upregulated. Therefore, we analyzed the proteins in the 3D models after 5 days of treatment. In response to IL-17A an increase in S100A7 (S100 calcium binding protein A7, aka Psoriasin), S100A8 (aka Calgranulin-A), S100A9 (aka Calgranulin-B), S100A7A (aka S100A15 or koebnerisin), and hBD-2 (human beta-defensin 2 or human beta-defensin 4A) was measured by immunofluorescence, Western blotting and/or ELISA, dependent on the reactivities of the antibodies that were available (Fig. 1b–d). Moreover, we observed that IL-17A induced the expression of IL-36γ in the 3D model (Fig. 1c). These findings supported the gene expression analysis in response to IL-17A.

We also treated explants of human skin with different concentrations of IL-17A for 1, 2 and 4 days. We observed an increase in S100A7/psoriasin expression (Fig. 1e and f, and Supplementary Fig. S2a), a protein that controls cell proliferation and differentiation, has AMP activity, and promotes the inflammatory process in psoriasis^37^. We also analyzed filaggrin expression but were unable to observe differences, probably because of the long half-life of the protein in the more differentiated skin layers (Supplementary Fig. S2b). Similarly, involucrin, loricrin and keratin 10 expression did not change during 4 days of treatment (data not shown). Although we did not see any effect on these differentiation markers, the expression of the corresponding mRNAs was reduced in response to IL-17A (Supplementary Fig. S2c).

### IL-17A activates the anti-microbial barrier of the skin

The stimulation of AMPs suggested that the IL-17A-treated 3D models might be more efficient in interfering with bacterial growth compared to the untreated control. *Staphyloccocus aureus* with an integreated GFP transgene^38^ were applied to 5d old control and IL-17A-treated 3D models. Bacterial growth was assessed 24 h later by staining sections with an *S. aureus*-specific antibody (Fig. 1g) and by measuring both GFP DNA and mRNA extracted from these models (Fig. 1h and i). The IL-17A-treated models supported bacterial growth substantially less well than control models indicating functional relevance of the induced AMPs.

### IL-17A activates AMPs and IL-36 cytokines in keratinocyte monolayer cultures

**Figure 2:**
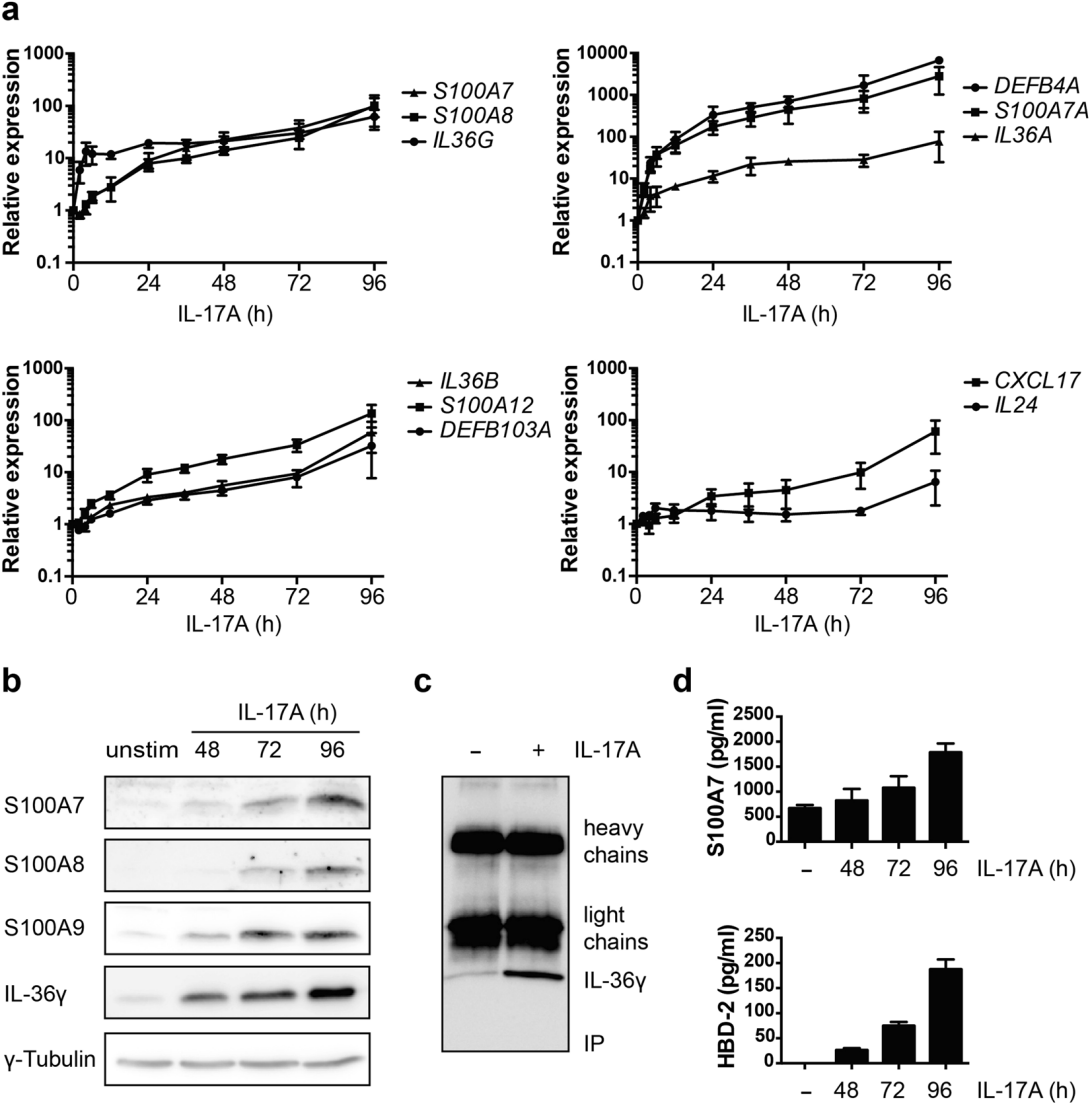
Regulation of gene expression by IL-17A in NHEK cells. (**a**) RNA analysis of the indicated genes in NHEKs stimulated with IL-17A for the indicated times (1 h–96 h). The mean values ± SD of three independent experiments are shown. (**b**) NHEKs were stimulated with IL-17A as indicated. RIPA cell lysates were analyzed for AMPs and IL-36γ by Western blotting. The entire blots are displayed in Supplementary Fig. S10b. (**c**) NHEKs were stimulated with or without IL-17A (50 ng/ml) for 96 h. The supernatants were harvested and IL-36γ was immunoprecipitated and subsequently detected on Western blots. (**d**) The supernatants of NHEKs stimulated with IL-17A as indicated were analyzed for S100A7 and hBD-2 by ELISA. The mean values ± SD of two independent experiments measured in duplicates are shown.

To study IL-17A signaling in more detail, we measured its effects in NHEK monolayer cultures. Our previous studies with IL-31 had shown substantial differences in gene expression when 3D models and monolayer were compared^39,40^. In contrast to these findings, all genes tested could be verified in NHEK monolayers, including the AMP encoding genes *S100A7*, *S100A7A*, *S100A8*, *S100A12*, *DEFB103A* and *DEFB4A*, and the cytokine/chemokine genes *IL36A*, *IL36B*, *IL36G*, *CXCL17*, and *IL24* (Fig. 2a). The activation of some of these genes, including *DEFB4A* and *S100A7A*, was continuously increasing over the entire time course, reaching several hundred fold after 96 hours (Fig. 2a). The expression of the encoded proteins, as far as suitable antibodies were available, were also enhanced (Fig. 2b–d). Of note was that IL-36γ was accumulating in the supernatant of IL-17A-stimulated NHEKs (Fig. 2c). Thus, IL-17A activated genes encoding AMPs and IL-36 family members in both 3D models and in NHEK monolayer cultures.

### Cytokines of the IL-36 family interfere with keratinocyte differentiation

**Figure 3:**
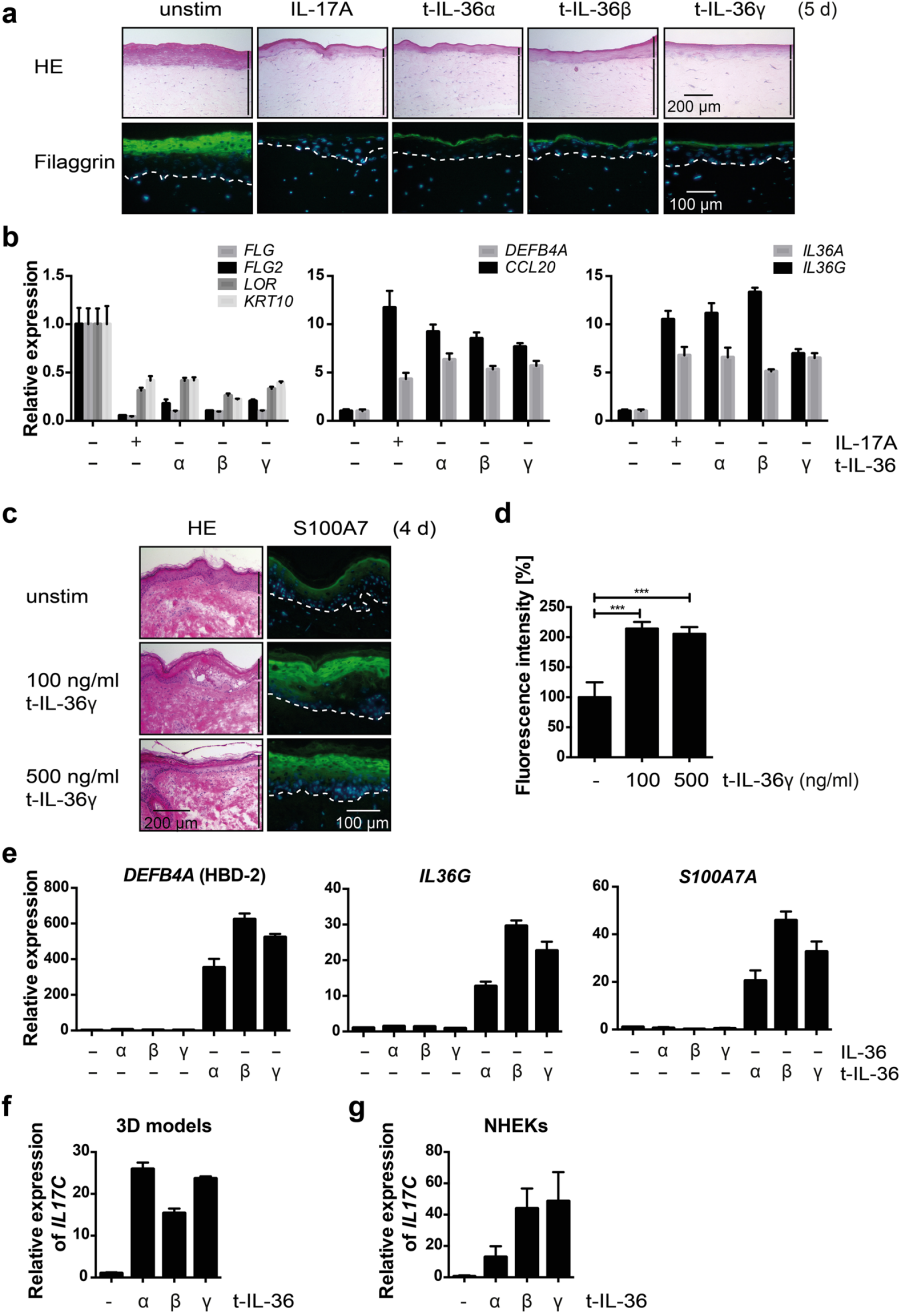
N-terminally truncated and activated IL-36 cytokines interfere with keratinocyte differentiation. (**a**) NHEK control 3D models were stimulated with or without IL-17A (50 ng/ml), t-IL-36α, t-IL-36β or t-IL-36γ (100 ng/ml each) for 5 d. The models were stained with H&E and for filaggrin (green), DNA is blue. (**b**) RNA was isolated from 3D models shown in panel (a) and the indicated genes were analyzed using qRT-PCR. The mean values ± SD of three technical replicates are shown. (**c**) Human *ex vivo* skin explants were cultivated with and without 100 or 500 ng/ml t-IL-36γ and harvested 4 days later. Histological sections were stained for S100A7 (green), DNA is blue. (**d**) Quantification of S100A7 fluorescence intensity of three sections of the *ex vivo* models shown in panel (c). ***p < 0.001. (**e**) NHEKs were stimulated with full length IL-36α, β and γ or with the truncated variants t-IL-36α, β and γ of these cytokines for 18 h. RNA was isolated and qRT-PCR analysis of the indicated genes performed. The mean values ± SD of one representative experiment measured in triplicates is shown. Two additional experiments are shown in Supplementary Fig. S3a. (**f**) qRT-PCR analysis of *IL17C* of an NHEK 3D model stimulated with or without t-IL-36α, β and γ (100 ng/ml each). The mean values ± SD of one experiment measured in triplicates is shown. (**g**) qRT-PCR analysis of *IL17C* of NHEKs stimulated with or without t-IL-36α, β and γ (100 ng/ml each) for 18 h. The mean values ± SD of one experiment measured in triplicates is shown. Two bars on the right side of the H&E stainings indicate epidermis (upper bar) and dermis (lower bar) in the skin models. In the immunofluorescent stainings the basal layer is marked with a dotted line.

Of particular interest to us was the induction of mRNAs and proteins of *IL36* genes. The expression of IL-36 cytokines is elevated in many inflammatory diseases^22,41^. In particular, high IL-36 as well as mutations in IL-36 signaling components are associated with psoriasis^25,30,42–45^. The findings that IL-17A induced the expression of the IL-36 cytokines suggested that these are downstream effectors of IL-17A. Therefore, we tested whether different IL-36 family members affected keratinocyte differentiation in the 3D model. N-terminally processed and activated forms of IL-36α, β and γ (t-IL-36 for N-terminally truncated) prevented efficient differentiation and reduced filaggrin expression (Fig. 3a). Consistent with this observation, the expression of various epidermal differentiation markers, including *FLG*, *FLG2*, *LOR*, and *KRT10*, was repressed by all three t-IL-36 cytokines, similar to the response to IL-17A (Fig. 3b). Moreover, the expression of *DEFB4A* and *CCL20*, the latter encodes a factor relevant for neutrophil migration, and of *IL36A* and *IL36G* were activated comparably to IL-17A (Fig. 3b). In addition, t-IL-36γ also induced the AMP S100A7 in skin explants after 1, 2 and 4 days (Fig. 3c and d, Supplementary Fig. 2a). As for the analysis of IL-17A, filaggrin and other differentiation markers were not affected within this time frame (Supplementary Fig. S2b, and data not shown). Nevertheless, the mRNA expression of these differentiation markers was reduced in response to IL-36γ (Supplementary Fig. S2c). Thus, activated IL-36 cytokines had, as far as measured, comparable effects to IL-17A in 3D models and human skin explants.

In NHEK monolayer cultures *DEFB4A*, *IL36G*, and *S100A7A* were induced by the processed, active forms of all three IL-36 family members, but not the full-length proteins (Fig. 3e and Supplementary Fig. 3a). Because IL-17A stimulated IL-36 cytokine expression, it is possible that these cytokines cooperate in feedback loops. We observed that t-IL-36α, β and γ induced *IL17C* expression, as did IL-17A, both in 3D models and in NHEK monolayer cultures (Fig. 3f and g and Supplementary Fig. 3b and c). Together our results suggest that IL-36 family members have similar effects on 3D organotypic skin models and NHEK monolayer cultures as IL-17A and thus appear to be candidates to promote and enhance the consequences of IL-17A in the skin.

### Keratinocytes secrete IL-36 in a latent form requiring activation by proteases

**Figure 4:**
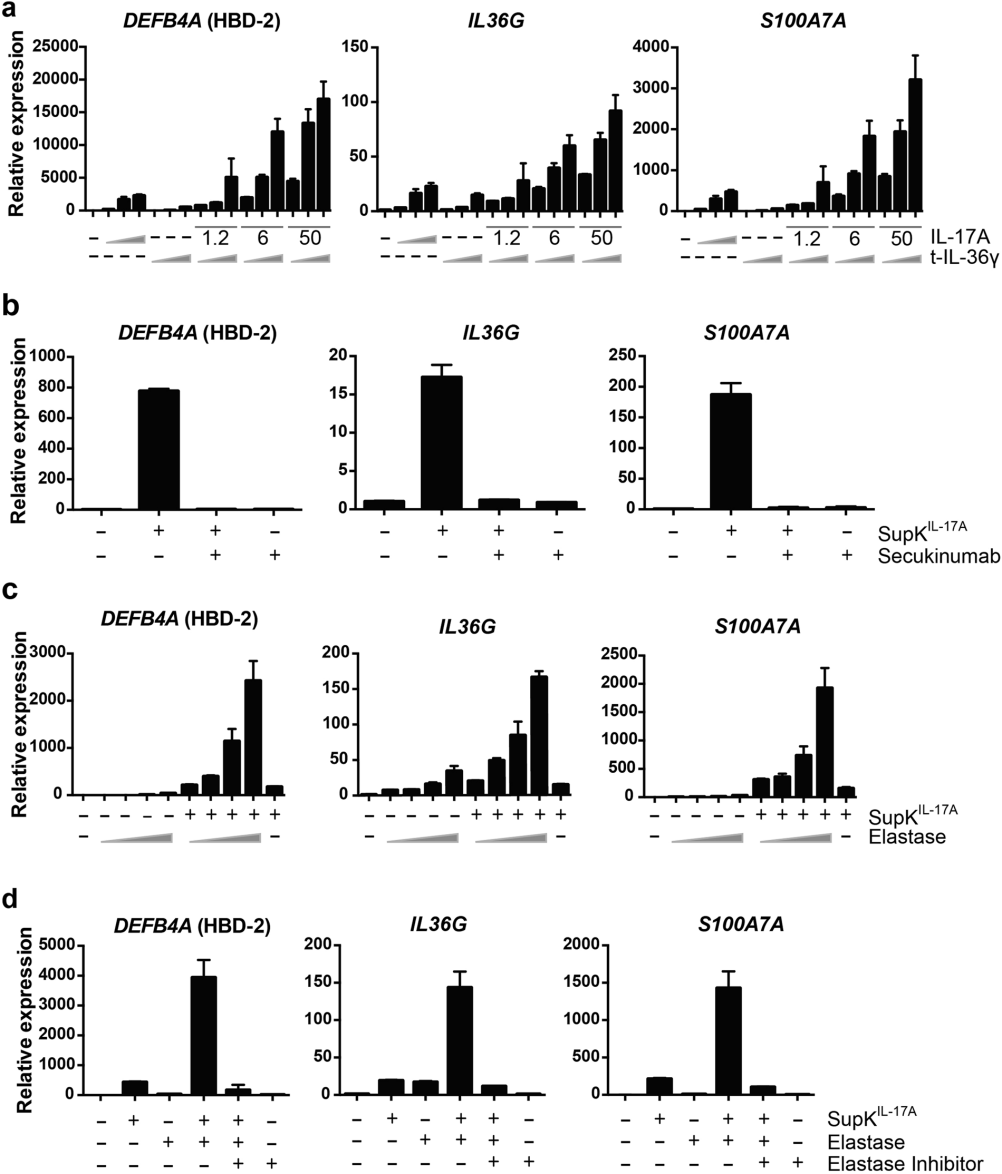
Target genes are activated cooperatively by IL-17A and t-IL36γ in NHEK monolayer cultures. (**a**) NHEKs were stimulated with different concentrations of IL-17A (1.2, 6 and 50 ng/ml) or t-IL36 γ (2.4, 12 and 100 ng/ml) alone or in combination for 48 h. RNA was isolated, cDNA produced and qRT-PCR was performed for the indicated genes. The mean values ± SD of one representative experiment measured in triplicates are shown. An additional experiment is shown in Supplementary Fig. S4a. (**b**) NHEKs were incubated with supernatant of NHEKs stimulated with IL-17A for 96 h diluted 1:2 with or without the addition of Secukinumab (4.3 μg/ml) for 16 h. (**c**) The supernatants of NHEKs stimulated with IL-17A for 96 h were incubated with different amounts of elastase (10, 20, 30 and 50 nM) for 2 h at 37 °C. The mixtures were diluted 1:2 and then NHEKs were incubated with these samples for 24 h. One experiment measured in duplicates is shown. (**d**) The supernatant of NHEKs stimulated with IL-17A for 96 h was incubated with elastase (20 nM) and elastase inhibitor (α1-antitrypsin, 800 nM) as indicated for 2 h at 37 °C. These mixtures were diluted 1:2 and then added to naïve NHEKs for 16 h. The mean values ± SD of one representative experiment measured in duplicates is shown. Two additional experiments are shown in Supplementary Fig. S7a. SupK^IL-17A^ indicates supernatant of NHEK cells stimulated with IL-17A.

From the analyses described above, we speculated that IL-17A and t-IL-36γ might cooperate in inducing the *DEFB4A*, *IL36G*, and *S100A7A* target genes. Indeed, we observed that IL-17A and t-IL-36γ activated these genes synergistically (Fig. 4a and Supplementary Fig. S4a). Similarly, t-IL-36α and β synergized with IL-17A (Supplementary Fig. 4b–e). These observations motivated us to test whether supernatants of IL-17A-treated NHEKs, which contain IL-36γ (Fig. 2c), were more active in stimulating *DEFB4A*, *IL36G*, and *S100A7A* than IL-17A alone. However, the activity of supernatants of IL-17A-treated NHEK cells, harvested 96 h after stimulation, on naïve NHEKs was completely blocked by Secukinumab (Fig. 4b), which inhibited all IL-17A effects both in 3D models and in NHEK monolayer cultures (Supplementary Fig. S5). Of note is also that Secukinumab did not interfer with IL-36 signaling (Supplementary Fig. S6). This indicated that sufficient IL-17A was still present in these supernatants that stimulated gene expression. As mentioned above, IL-36 cytokines require processing to gain biological activity^34–36^. IL-36 can be processed and activated by proteases derived from neutrophils^25,36^. Elastase, one of these proteases, was capable of activating *DEFB4A*, *IL36G*, and *S100A7A* gene expression in naïve NHEK monolayer cultures cooperatively with IL-17A-treated NHEK supernatants (Fig. 4c and d and Supplementary Fig. S7a). We note that elastase stimulated weak gene expression in the absence of conditioned supernatants, possibly by activating a membrane-associated signal (Fig. 4c). The specificity of the effect was demonstrated by employing an elastase inhibitor, α1-antitrypsin, which blocked the protease effect (Fig. 4d and Supplementary Fig. S7a). As shown previously, recombinant IL-36γ was activated by recombinant elastase and by supernatants of activated neutrophils (Supplementary Fig. S7b and c)^36^.

**Figure 5:**
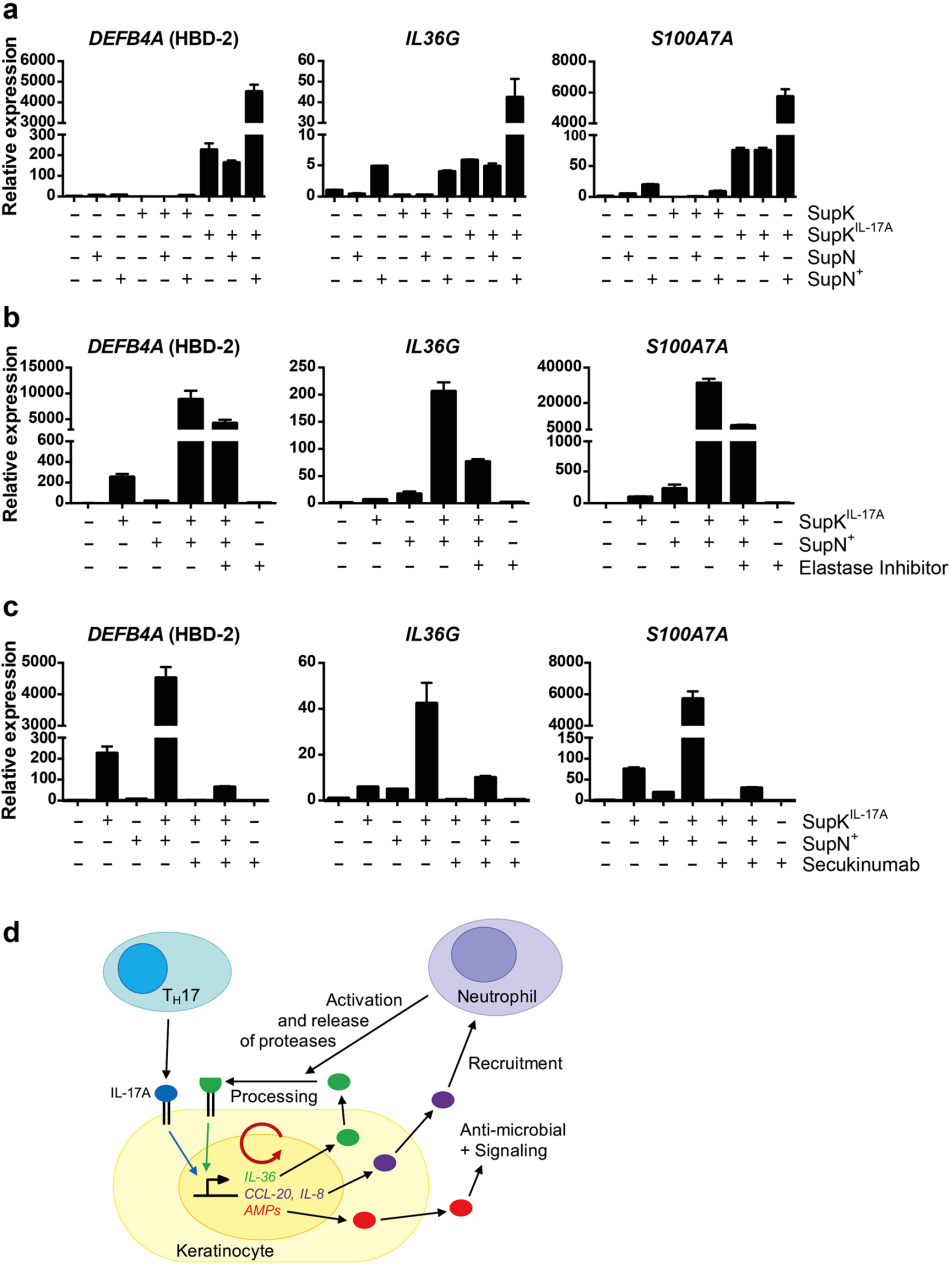
The supernatants of degranulated neutrophils activate IL-36 in NHEK supernatants. (**a**) The supernatants of NHEKs stimulated with or without IL-17A (50 ng/ml) for 96 h were incubated with supernatants of neutrophils, which were pretreated with or without PMA (SupN or SupN^+^, respectively), for 2 h at 37 °C. The mixtures were diluted 1:2 and then added to naïve NHEKs for 16 h. The mean values ± SD of one representative experiment measured in triplicates are shown. One additional experiment is shown in Supplementary Fig. S8a. (**b**) The supernatants of NHEKs stimulated with IL-17A for 96 h were incubated with or without supernatant of PMA-treated neutrophils and elastase inhibitor (800 nM) for 2 h at 37 °C. The mixtures were diluted 1:2 and then naïve NHEKs were incubated with these samples for 16 h. One experiment measured in triplicates is shown. (**c**) The supernatants of NHEKs stimulated with IL-17A for 96 h were incubated with or without supernatant of PMA-treated neutrophils and Secukinumab (4.3 μg/ml) for 2 h at 37 °C. The mixtures were diluted 1:2 and then naïve NHEKs were incubated with these mixtures for 16 h. The mean values ± SD of one representative experiment measured in triplicates is shown. Two additional experiments are shown in Supplementary Fig. S9. (**d**) Schematic summary of our findings indicating that IL-17A controls genes that encode cytokines and AMPs, among others. These participate in the chemical barrier and control anti-microbial activity and various signaling events, including crosstalk with immune cells, such as attracting neutrophils, and feedback control of keratinocytes. The latter comprises IL-36 cytokines that need to be activated by proteolytic processing, e.g. by neutrophil proteases, and cooperate with IL-17A. Not shown are the IL-17A and IL-36 target genes, whose products are contributing to the deregulated keratinocyte differentiation and are associated with the psoriatic phenotype. SupK and SupK^IL-17A^ indicates supernatant of NHEK cells stimulated without or with IL-17A, respectively. SupN and SupN^+^ indicates supernatant of primary neutrophils treated without or with PMA, respectively.

Next, we combined supernatants of NHEK monolayer cultures, either untreated or IL-17A-stimulated, and supernatants of neutrophils, either control or phorbol myristate acetate (PMA)-treated, to stimulate naïve NHEK cultures. The analysis of *DEFB4A*, *IL36G*, and *S100A7A* demonstrated a strong synergism of the supernatants of IL-17A-treated NHEKs and PMA-stimulated neutrophils (Fig. 5a and Supplementary Fig. S8). This synergism was reduced in the presence of α1-antitrypsin, which inhibits in addition to elastase also cathepsin G and proteinase 3, supporting the notion that these proteases are important for activating IL-36 cytokines (Fig. 5b). PMA on its own did not synergize with supernatants of IL-17A-stimulated NHEK monolayer cultures (data not shown). α1-antitrypsin was not fully repressing the cooperative effects suggesting that these are not the only neutrophilic proteases involved. IL-36α and β are expressed in the NHEKs in response to IL-17A and might also contribute to the cooperative effects, particularly as they are activated by distinct proteases^25,36^. The importance of neutrophil-derived proteases was further demonstrated by the activation of latent IL-36γ in the presence of neutrophil supernatants or by elastase as measured by *DEFB4A* and *S100A7A* expression (Supplementary Fig. S7b and c). This activation was inhibited in the presence of a protease inhibitor, supporting the notion that neutrophil-derived proteases are critical for converting latent IL-36γ into its signaling competent form. Finally, we combined the supernatants of IL-17A-treated NHEK cells and of PMA-treated neutrophils and blocked the remaining IL-17A with Secukinumab to measure IL-17A independent signaling. This revealed weak activity suggesting that the amounts of IL-36 released and/or the protease activity in the neutrophil supernatants were rather low (Fig. 5c and Supplementary Fig. S9). Nevertheless, these findings support the concept that keratinocytes produce and secrete latent IL-36 cytokines, which upon processing and activation, for example by proteases released from neutrophils, cooperate efficiently with IL-17A.

## Discussion

Psoriasis is associated with systemic inflammatory processes that affect the differentiation of keratinocytes with the IL-23 – IL-17 axis playing an important role in disease progression^6^. We have used 3D organotypic skin equivalents to demonstrate that IL-17A interferes with keratinocyte differentiation and deregulates gene expression associated with skin development and differentiation, and with signaling processes (Figs 1–2 and Supplementary Fig. S1). Of particular interest to us were genes encoding AMPs and IL-36 family members. The enhanced expression of AMPs reduced proliferation of *S. aureus* inoculated onto 3D models (Fig. 1g–i). The induction of IL-36-type cytokines suggested that these are potential downstream effectors of IL-17A and relevant for the psoriasis phenotype, as has been suggested before^23^. IL-36 cytokines that are induced in and secreted by NHEKs in response to IL-17A are inactive (Fig. 2), most likely because of lack of N-terminal processing^35^. In agreement with previous findings^25,36^, IL-36 can be activated by proteases that are released by activated neutrophils, including elastase (Supplementary Fig. S7). Not only that IL-36 is induced and secreted in response to IL-17A, the activated forms stimulate NHEKs cooperatively with IL-17A (Fig. 4 and Supplementary Fig. S4). Finally, IL-36 cytokines can also stimulate the expression of *IL17C* (Fig. 3f and g). Thus, these findings suggest that the interaction of T cells, neutrophils and keratinocytes, and possibly other cell types, communicate through a network of cytokines and proteases, with IL-17 and IL-36 playing a prominent role. They cooperate in activating target genes in keratinocytes in a potentially iterative manner. Thus our findings provide evidence for a vicious cycle of cytokine-mediated disease progression that is blocked by interfering with IL-17A, as clinically employed with Secukinumab, and offers additional entry points for interference (Fig. 5d). One of the side effects of interfering with IL-17A signaling is the occurrence of *Candida albicans* infections, consistent with the important role of the IL-17 family cytokines in protecting the skin and mucous membranes against *Candida* ^46^. Thus, targeting downstream effectors of IL-17A such as IL-36 cytokines and their regulators might be clinically interesting.

Our IL-17-driven gene expression studies in 3D models support and expand previous analysis and define genes associated with keratinocyte differentiation, anti-microbial defense and immunmodulation as important targets (Fig. 1 and Supplementary Fig. S1)^16–20,30^. In agreement, we find that IL-17A interferes with differentiation and alters gene expression, which is completely blocked by Secukinumab (Supplementary Fig. S5)^14,15^. The functionally relevant induction of AMPs is consistent with studies in other tissues that also reported the induction of AMPs in response to IL-17 cytokines^47–49^. Of note is that AMPs also possess signaling functions for various cell types^50^. Thus, the induction of AMPs might be relevant for controling immune cells function together with for example CCL-20 and IL-8, in addition to affecting microbial growth and survival (Fig. 1g–i), an aspect that will be interesting to address. Together, these and other studies provide a mechanistic explanation for the differentiation defect of keratinocytes observed in psoriatic lesions. Moreover, these factors may contribute to the recruitment of immune cells, including neutrophils, which can further aggravate the inflammatory progression of psoriatic lesions through a crosstalk between keratinocytes and immune cells^21^.

Besides IL-17 and cytokines such as IL-12 and IL-23^6^, IL-36 cytokines are important in psoriasis^24–30^. This is supported by our observations that IL-36 cytokines are induced in response to IL-17A in NHEKs, both in 3D models and in monolayer cultures, and that IL-36 inhibited keratinocyte differentiation in 3D models (Figs 1–3 and Supplementary Fig. 1). In addition, IL-36 cytokines control a number of different inflammatory processes associated not only with the skin but also with the lung and the central nervous system among others^22,23,51^. IL-36 cytokines belong to the IL-1 family and interact with receptor dimers composed of IL-36R and IL-1RAcP, the latter being a receptor subunit used also by other IL-1 family cytokines^23,52^. Like other IL-1 family members, IL-36 cytokines need to be processed to achieve full biological activity (Fig. 3e and Supplementary Fig. S3a)^25,34–36,53^. Together, these observations suggest that IL-36 and its processing factors are likely to be important in psoriasis.

Recent studies have shed light onto how IL-36 cytokines are processed and activated. It was noted that removing N-terminal amino acids increased their signaling capacity^35^. New results describe neutrophil-derived proteases as well as cathespsin S, a lysosomal protease, as IL-36 cytokine processing enzymes^25,34,36^. In both situations the cleavage and activation of IL-36 appears to occur extracellularly, unlike other IL-1 family members that are primarily processed intracellularly, prior to secretion by non-conventional mechanisms^53^. We confirmed the ability of neutrophil-derived proteases, including elastase, to activate IL-36γ (Supplementary Fig. S7b and c). These findings were expanded by demonstrating that IL-36 cytokines are released from keratinocytes in response to IL-17A stimulation but are unable to induce target gene expression. It required protease activity of activated neutrophils (Figs 2, 4 and 5, Supplementary Figs S4 and 7–9). So far we have no evidence that keratinocyte-derived proteases can activate latent IL-36 cytokines in our experiments.

Of note was the strong synergism between activated IL-36 cytokines and IL-17A, which we observed with purified factors and with protease-treated supernatants (Figs 4 and 5 and Supplementary Figs S4 and 7–9). For example, the mRNA expression of *S100A7A*, which was stimulated roughly 40- and 50-fold by 1.2 ng/ml IL-17A and 100 ng/ml t-IL-36γ, respectively, was activated more than 700-fold when the two cytokines were combined (Fig. 4). Thus, we propose that IL-17A activates genes whose products control molecular signaling pathways that reinforce IL-17A signaling. These include factors that have the potential to attract additional immune cells, including neutrophils. Their stimulation, e.g. in response to pathogen-associated molecular patterns (PAMPs), can release IL-36 processing proteases. Subsequently, the activated IL-36 cytokines cooperate with the initial IL-17A signal (Fig. 5d). This suggests an amplifying mechanism promoting skin inflammation and persistent psoriatic symptoms.

## Methods

### Primary cell culture and organotypic 3D skin equivalents

Normal human epidermal keratinocytes (NHEKs) and human dermal fibroblasts (HDFs) were prepared from sterile human skin samples of healthy individuals. Informed consent was obtained from all subjects in accordance with the procedure that was approved by the ethic committee of the Medical School of the RWTH Aachen University (Certificate No EK 188/14 and EK 130/15). The cells were cultivated under standard cell culture conditions as described previously^54^. Organotypic 3 dimensional skin equivalents were constructed as described previously^39,54^, cultured at the air-liquid interphase, and treated with recombinant human (rh) IL-17A (Peprotech, Hamburg, Germany), truncated (t-)IL-36α/IL-1F6 (aa 6-158), t-IL-36β/IL-1F8 (aa 5-157) and t-IL-36γ/IL-1F9 (aa 18-169) (R&D, Minneapolis, USA). Secukinumab (aka Cosentyx®, Novartis) was used as indicated in the figure legends, but typically at a concentration of 4.3 μg/ml, which correlates with the concentration used for clinical treatment (300 mg per day per patient (70 kg)). During cultivation the medium was changed every second day. The skin equivalents were harvested, one part was embedded in Tissue Tec (O.C.T.) compound (Sakura Finetek, Zoeterwoude, The Netherlands) for cryosectioning, the other was stored in RNA later (Ambion/Applied Biosystems, Darmstadt, Germany) for subsequent RNA isolation. Two dimensional NHEK cultures were treated with or without IL-17A, IL-36α/IL-1F6, IL-36β/IL-1F8, IL-36γ/IL-1F9, t-IL-36α/IL-1F6 (aa 6-158), t-IL-36β/IL-1F8 (aa 5-157) or t-IL-36γ/IL-1F9 (aa 18-169) as indicated in the figure legends. All methods were carried out in accordance with relevant guidelines and regulations (approved by the ethic committee of the Medical School of the RWTH Aachen University).

### *Ex vivo* skin explants

*Ex vivo* skin explants were prepared from sterile human skin samples (approved by the ethics committee of the Medical School of the RWTH Aachen University (Certificate No EK 065/16)) as described previously^40^. In brief, eight-millimeter punch biopsies were taken from these skin samples and mounted on polycarbonate membrane inserts (Nunc, Rochester, NY), which were placed in six-well plates. Skin grafts were cultured at the air–liquid interphase and cultivated under standard cell culture conditions. The explants were maintained with and without 50 or 500 ng/ml IL-17A (PeproTech) or 100 or 500 ng/ml t-IL-36γ (R&D) and the medium was changed daily. After 4 d the explants were harvested as described for organotypic 3D skin equivalents.

### Exon expression arrays

For gene expression analysis, NHEK organotypic 3D skin equivalents were stimulated with IL-17A (50 ng/ml, PeproTech) for 24 h or 48 h. Then mRNA was isolated, purified and analyzed on GeneChip Human Exon 2.0 ST arrays as reported previously^39,40^. Data analysis was performed with GeneSpring GX 11 software (Agilent, Böblingen, Germany). Gene ontology analysis was generated using http://www.geneontology.org/.

### Western Blotting

NHEKs or 3D models were lysed in RIPA buffer (10 mM Tris/HCl, pH 7.4, 150 mM NaCl, 1% Nonidet P-40, 1% deoxycholic acid, 0.1% SDS, 0.5% Trasylol) containing a protease inhibitor cocktail (Sigma-Aldrich), on ice. In case of organotypic 3D models, the tissues were mechanically disrupted and homogenized by using tissue lyzer (Qiagen). The lysates were sonicated for 15 min in a water bath sonicater and cleared by centrifugation for 30 min, 13.200 rpm at 4 °C in an Eppendorf centrifuge. Proteins were separated using 12–20% SDS-PAGE and then blotted on nitrocellulose membranes. For the detection of different proteins specific antibodies were purchased from abcam (S100A7 (ab13680), S100A9 (ab92507)), R&D (IL-36γ (AF2320)), Santa Cruz Biotechnology (S100A8 (sc-20174)), and Sigma (γ-tubulin (T-6557)).

### RNA preparation, reverse transcription, and quantitative RT-PCR

Nucleo Spin RNA II (Macherey-Nagel, Düren, Germany) kits were used for total RNA extraction, according to the manufacturer’s instruction, and residual genomic DNA was removed by rDNase (RNase-free) digestion. The tissues of organotypic 3D models were mechanically disrupted and homogenized by using tissue lyzer (Qiagen). A total of 1 µg of RNA was reverse transcribed into cDNA by utilizing High Capacity RNA-to-cDNA Master Mix (Applied Biosystems, Foster City, CA, USA). TaqMan experiments were carried out on an ABI PRISM 7300 sequence detection system (Applied Biosystems) using Assay-on-Demand gene expression products (Applied Biosystems) for *CCL20* (Hs01011368_m1), *CXCL5* (Hs00171085_m1), *CXCL17* (Hs01650998_m1), *DEFB103A* (Hs00218678_m1), *DEFB4A* (Hs00823638_m1), *FLG* (Hs00856927_g1), *FLG2* (Hs00418578_m1), *GFP* (Mr03989638_mr), *IL1B* (Hs00174097_m1), *IL8* (Hs00174103_m1), *IL17C* (Hs00171163_m1), *IL36A* (Hs00205367_m1), *IL36B* (Hs00758166_m1), *IL36G* (Hs00219742_m1), *IL36RN* (Hs00202179_m1), *KRT10* (Hs00166289_m1), *LOR* (Hs01894962_m1), *S100A7* (Hs00161488_m1), *S100A7A* (Hs00752780_m1), *S100A8* (Hs00374263_m1) and *S100A12* (Hs00194525_m1) according to the manufacturer’s recommendations. An Assay-on-Demand product for *HPRT* mRNA (Hs99999909_m1) was used as an internal reference to normalize the target transcripts. All measurements were performed in duplicates or triplicates in separate reaction wells as indicated in the figure legends.

### Light microscopy and immunofluorescence

For light microscopy and immunofluorescence analyses of the organotypic 3D skin models, 4 µm cryosections were processed as described previously^39,40,55^. The sections were stained with H&E or used for immunofluorescence. The following antibodies specific for the indicated proteins were used: filaggrin (sc-66192, Santa Cruz, CA, USA), S100A7 (ab13680, abcam, Cambridge, U.K.), S100A9 (ab92507, abcam) and S100A7A (TU München, Germany); the DNA was stained with DAPI (Applichem, Darmstadt, Germany).

### Enzyme-linked immunosorbent assay (ELISA)

NHEKs were treated as described in the figure legends and supernatants were collected. The samples were used in ELISAs and measured in duplicates according to the manufacture’s recommendations (hBD-2: 900-K172, Peprotech, Hamburg Germany; S100A7: Cloud-Clone Corp, Houston TX, USA).

### Analysis of anti-microbial activity

The anti-microbial activity was analyzed with some modifications as described previously^40^. In brief, NHEK organotypic 3D skin equivalents were stimulated with or without rhIL-17A (50 ng/ml) for 5 d. *Staphylococcus aureus* stably expressing GFP (ATCC29213)^38^ was applied topically onto the skin equivalents. The models were harvested directly (0 h) or incubated at 37 °C and 5% CO_2_ for 24 h. The models were cut into three parts. One part was used for immunohistochemistry using an *S. aureus*-specific antibody (abcam, ab37644), the other two parts for isolation of genomic DNA or RNA. RNA was isolated according to the manufacturer’s instructions for Gram-positive bacteria and reverse transcribed into cDNA. Genomic DNA was prepared using the peqGOLD Bacterial DNA Kit (Peqlab). GFP DNA and cDNA was quantified by real-time PCR using the TaqMan system and an Assay-on-Demand gene expression product for *GFP* (Mr03989638_mr; Applied Biosystems). All measurements were performed in duplicates. For determining the relative bacterial growth, the changes of threshold cycle values for GFP from 0 to 24 h were calculated. The 0 h values were taken as background and subtracted from the 24 h values. The values given in Fig. 3 are 2DCT (i.e. fold change compared with the 0 h values in percent for each experiment). Values of control models were set as 1. This was compared to the treated models. Thus, relative bacterial growth after 24 h in the presence or absence of IL-17A is displayed upon subtracting the background. Five experiments were performed and analyzed for DNA and two experiments for RNA.

### Protease activity assays with neutrophil supernatants or elastase

Neutrophils were isolated and supernatants (degranulates) prepared as previously described^36^. Recombinant IL-36γ (75 ng/ml) or supernatants of NHEKs stimulated with or without IL-17A (50 ng/ml) for 96 h were incubated with elastase (the concentrations are indicated in the figure legends) or neutrophil supernatant +/− PMA stimulation for 2 h at 37 °C. These approaches were diluted 1:2 and then naïve NHEKs were incubated with these samples for 16–24 h. RNA was isolated, cDNA prepared and qRT-PCR performed as described.

### Statistics

Mann-Whitney test was used for statistical evaluation. *p < 0.05, **p < 0.01, ***p < 0.001.

### Data availability statement

The microarray datasets generated during and/or analyzed during the current study are available from the corresponding author on reasonable request.

## Electronic supplementary material

Supplementary PDF File
