# Supplementary material for: The psoriasis-associated IL-17A induces and cooperates with IL-36 cytokines to control keratinocyte differentiation and function
Source: Sci Rep. 2017 Nov 15;7:15631. doi: 10.1038/s41598-017-15892-7 (PMC5688102; doi:10.1038/s41598-017-15892-7)
Supplement: Supplementary file 1 — Supplementary PDF File [file 41598_2017_15892_MOESM1_ESM.pdf]

SUPPLEMENTARY DATA

The psoriasis-associated IL-17A induces and cooperates with IL-36 cytokines to control keratinocyte differentiation and function

Carolina M. Pfaff[1,2], Yvonne Marquardt[2], Katharina Fietkau[2], Jens M. Baron[2,*] and Bernhard Lüscher[1,*]

[1]Institute of Biochemistry and Molecular Biology and [2]Department of Dermatology and Allergology, Medical School, RWTH Aachen University, 52074 Aachen, Germany
[*]B.L. and J.M.B. are equally contributing senior authors.
ORCIDs: 0000-0002-9622-8709 (B.L.); 0000-0002-1174-6946 (J.M.B.).

a

		Fold induction			
		Model 1		Model 2	
		24 h	48 h	24 h	48 h
Cytokines/chemokines	*CCL20*	6.2	5.5	3.7	4.9
	IL8	5.3	2.8	1.6	2.8
	IL36A	111.6	77.5	32.5	44.0
	IL36B	4.0	6.8	4.4	3.7
	IL36G	13.6	82.4	11.8	12.0
Anti-microbial peptides	*S100A7A*	5.8	5.4	6.0	4.8
	S100A8	3.6	2.3	2.3	2.2
	DEFB4B	157.7	273.6	35.1	17.5
Differentiation markers	*KRT10*	0.6	0.0	0.9	0.6
	FLG	0.4	0.2	0.3	0.7

b

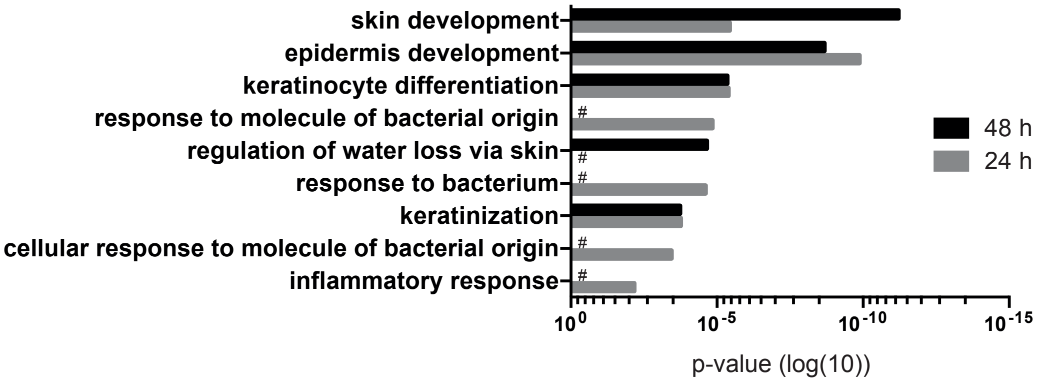

Supplementary Figure S1

Regulation of gene expression in response to the cytokine IL-17A in organotypic 3D skin equivalents.

(**a**) qRT-PCR analysis of two independent normal human epidermal keratinocytes (NHEK) 3D models stimulated with or without IL-17A for 24 h or 48 h.

(**b**) Gene ontology analysis of microarray results of NHEK 3D models stimulated with or without IL-17A for 24 h or 48 h with a cut-off value of 2.0. # not significant.

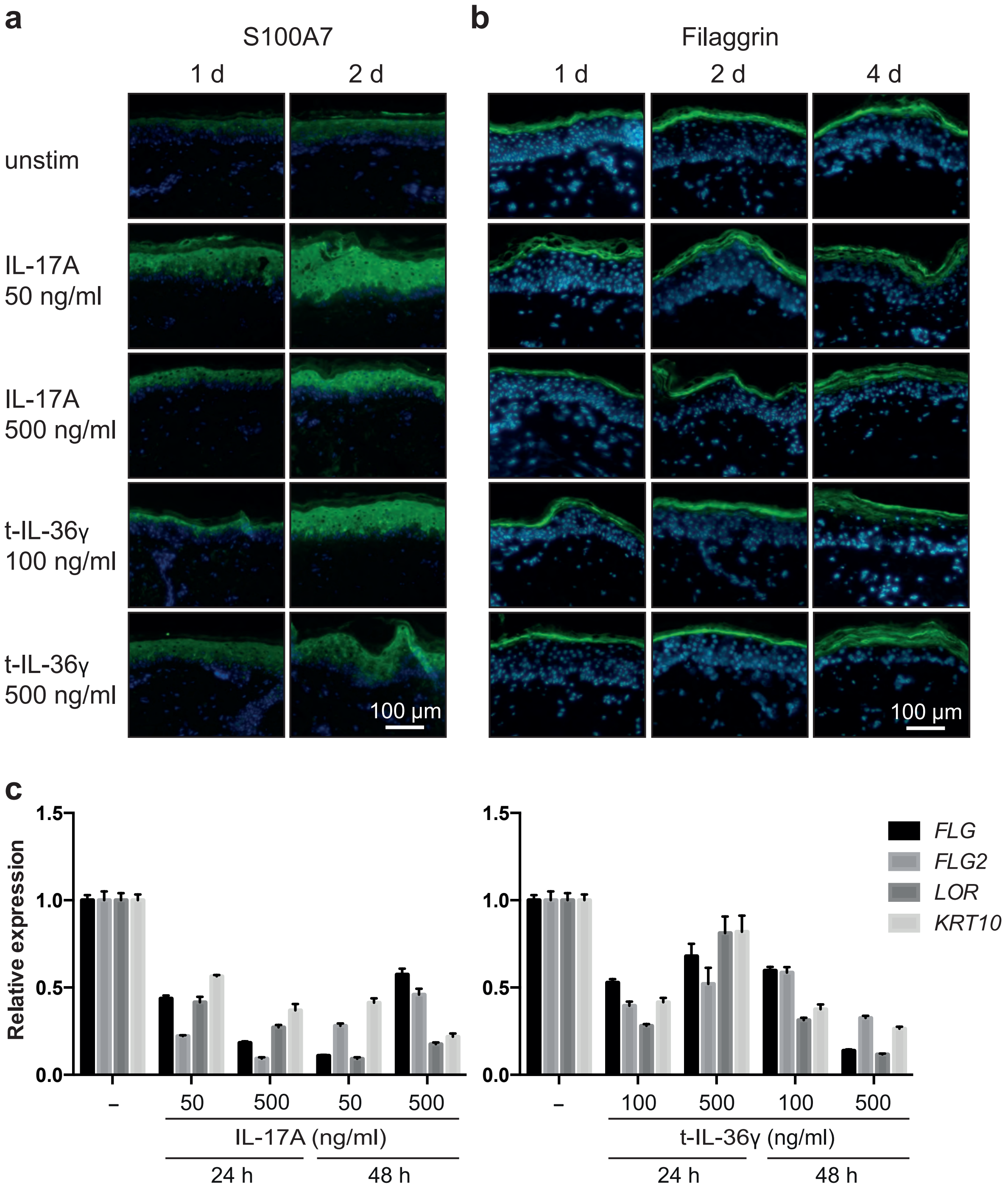

Supplementary Figure S2

IL-17A and N-terminally truncated IL-36γ regulate gene expression in skin explants.
Human *ex vivo* skin explants were cultivated with and without 50 or 500 ng/ml rhIL-17A or 100 or 500 ng/ml t-IL-36γ and harvested at the indicated time points.

(**a**) Histological sections were stained for S100A7 (green) at the indicated time points, the DNA is blue.

(**b**) Histological sections were stained for filaggrin (green) at the indicated time points, the DNA is blue.

(**c**) qRT-PCR analysis of the genes encoding filaggrin (*FLG*), filaggrin family member 2 (*FLG2*), loricrin (*LOR*), and cytokeratin 10 (*KRT10*) were analyzed in the skin explants treated as indicated.

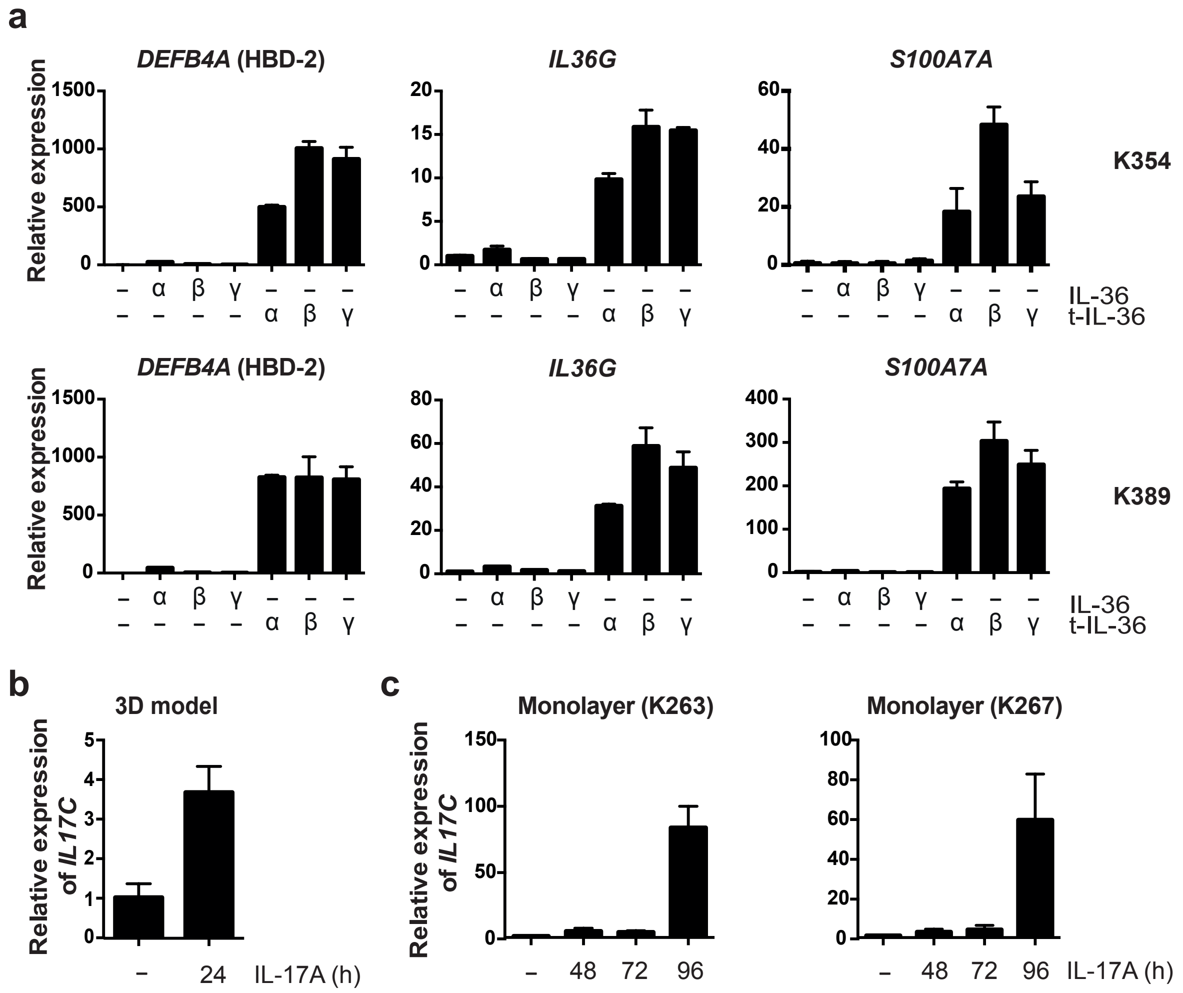

Supplementary Figure S3

IL-36 cytokines are only active in their N-terminally truncated forms.

(**a**) NHEKs monolayer cultures were stimulated with full-length IL-36α, β or γ or with the truncated and activated forms (indicated with t) of these cytokines (100 ng/ml) for 18 h. RNA was isolated and qRT-PCR analysis of the indicated genes performed. Two individual experiments with NHEK cells from two different donors measured in triplicates are shown.

(**b** and **c**) NHEK 3D skin equivalents (b) and 2 different NHEK monolayer cultures (c) were stimulated with or without IL-17A for the indicated times. The expression of *IL17C* was analyzed by qRT-PCR. The mean values ± SD of technical triplicates are shown in (b). The mean values ± SD of two biological replicates with technical duplicates are shown in (c).

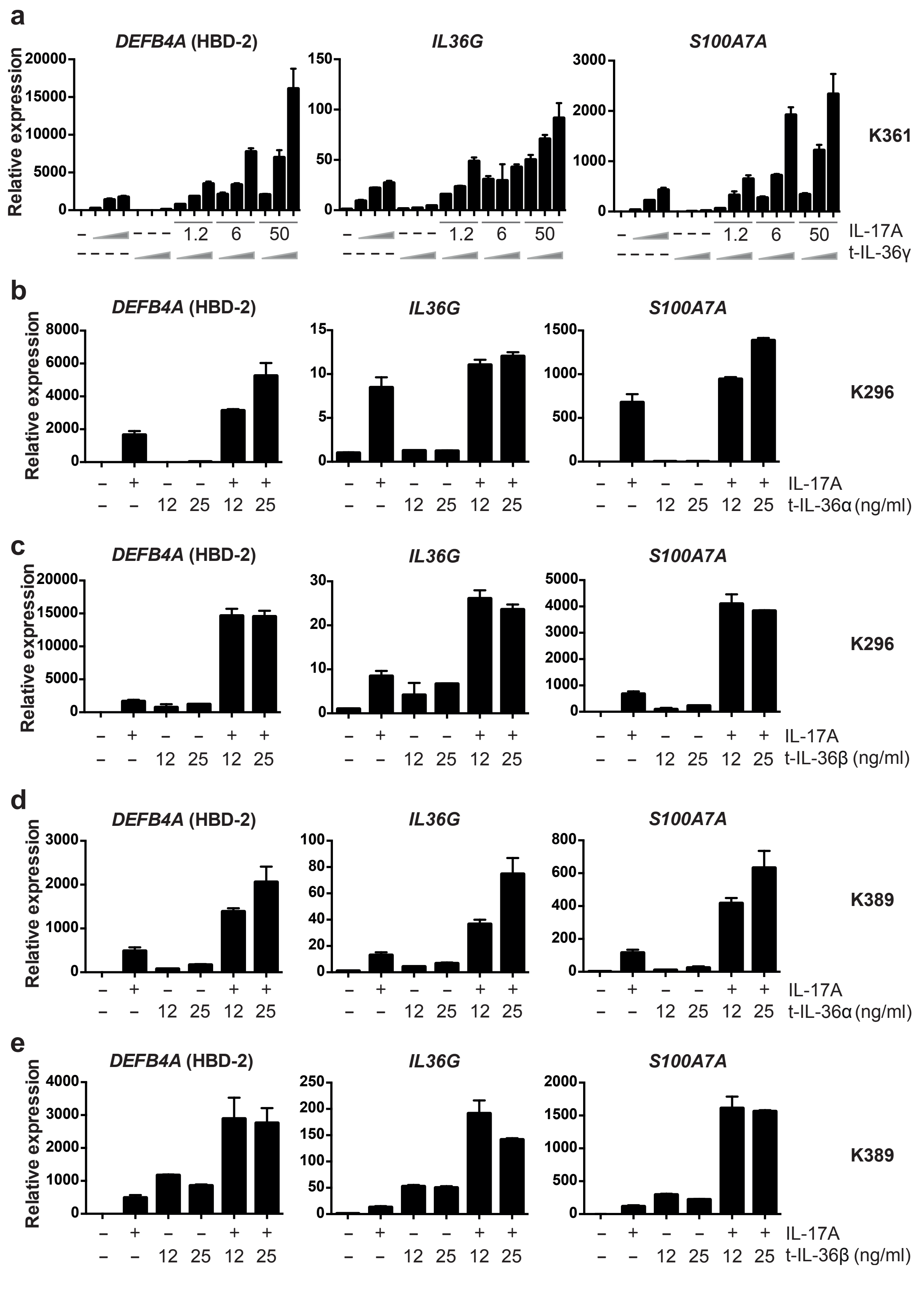

Supplementary Figure S4

IL-17A and IL-36 cooperate in the regulation of gene expression in NHEK cells.

(**a**) NHEKs were stimulated with different concentrations of IL-17A (1.2 ng/ml, 6 ng/ml and 50 ng/ml) and t-IL36γ (2.4, 12 and 100 ng/ml) as indicated alone or in combination for 48 h.

RNA was isolated, cDNA produced and the indicated genes were analyzed by qRT-PCR. The mean values ± SD of one representative experiment measured in triplicates is shown.

(**b**) NHEKs were stimulated with IL-17A (6 ng/ml) or t-IL36α (2.4, 12 and 100 ng/ml) as indicated alone or in combination for 48 h. RNA was isolated, cDNA produced and qRT-PCR was performed for the indicated genes. The mean values ± SD of one experiment with technical triplicates is shown.

(**c**) NHEKs were stimulated with IL-17A (6 ng/ml) or t-IL36β (2.4, 12 and 100 ng/ml) as indicated alone or in combination for 48 h. RNA was isolated, cDNA produced and qRT-PCR was performed for the indicated genes. The mean values ± SD of one experiment with technical triplicates is shown.

(**d**) NHEKs were stimulated with IL-17A (6 ng/ml) or t-IL36α (2.4, 12 and 100 ng/ml) as indicated alone or in combination for 18 h. RNA was isolated, cDNA produced and qRT-PCR was performed for the indicated genes. The mean values ± SD of one experiment with technical triplicates is shown.

(**e**) NHEKs were stimulated with IL-17A (6 ng/ml) or t-IL36β (2.4, 12 and 100 ng/ml) as indicated alone or in combination for 18 h. RNA was isolated, cDNA produced and qRT-PCR was performed for the indicated genes. The mean values ± SD of one experiment with technical triplicates is shown.

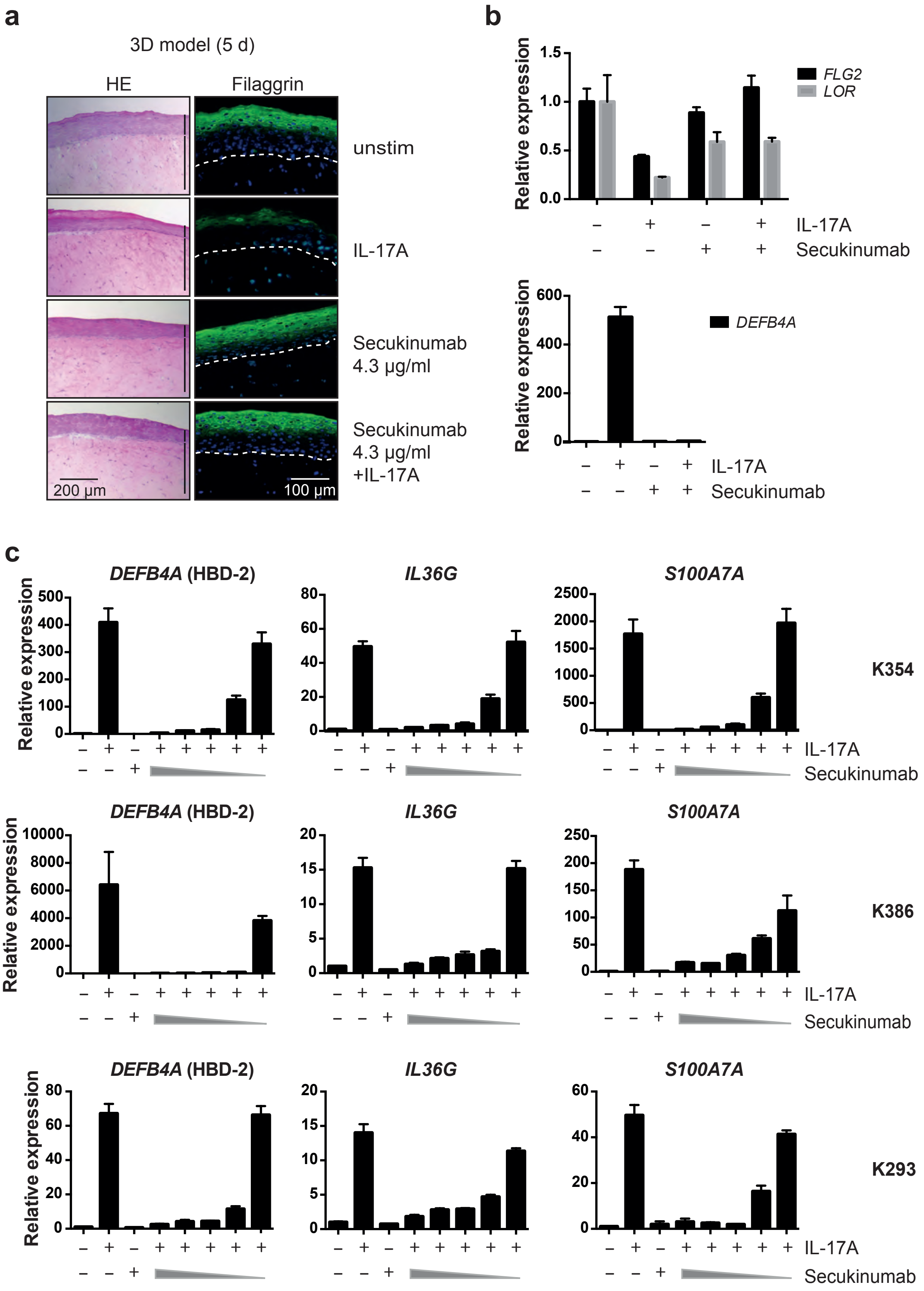

Supplementary Figure S5

The IL-17A-specific antibody Secukinumab blocks the IL-17A effects in 3D models and NHEKs.

(**a**) NHEK control 3D models stimulated with or without IL-17A and Secukinumab (4.3 µg/ml) for 5 days were stained with H&E and for filaggrin (green), DNA is blue. Two bars on the right side of the H&E stainings indicate epidermis (upper bar) and dermis (lower bar) in the skin models. In the immunofluorescent stainings the basal layer is marked with a dotted line.
(**b**) qRT-PCR analysis of the models shown in (a). The mean values ± SD of three technical replicates are shown.
(**c**) NHEKs stimulated with or without IL-17A and Secukinumab in different concentrations (43 ng/ml – 4.3 µg/ml) for 48 h. Three individual experiments with technical triplicates are shown.

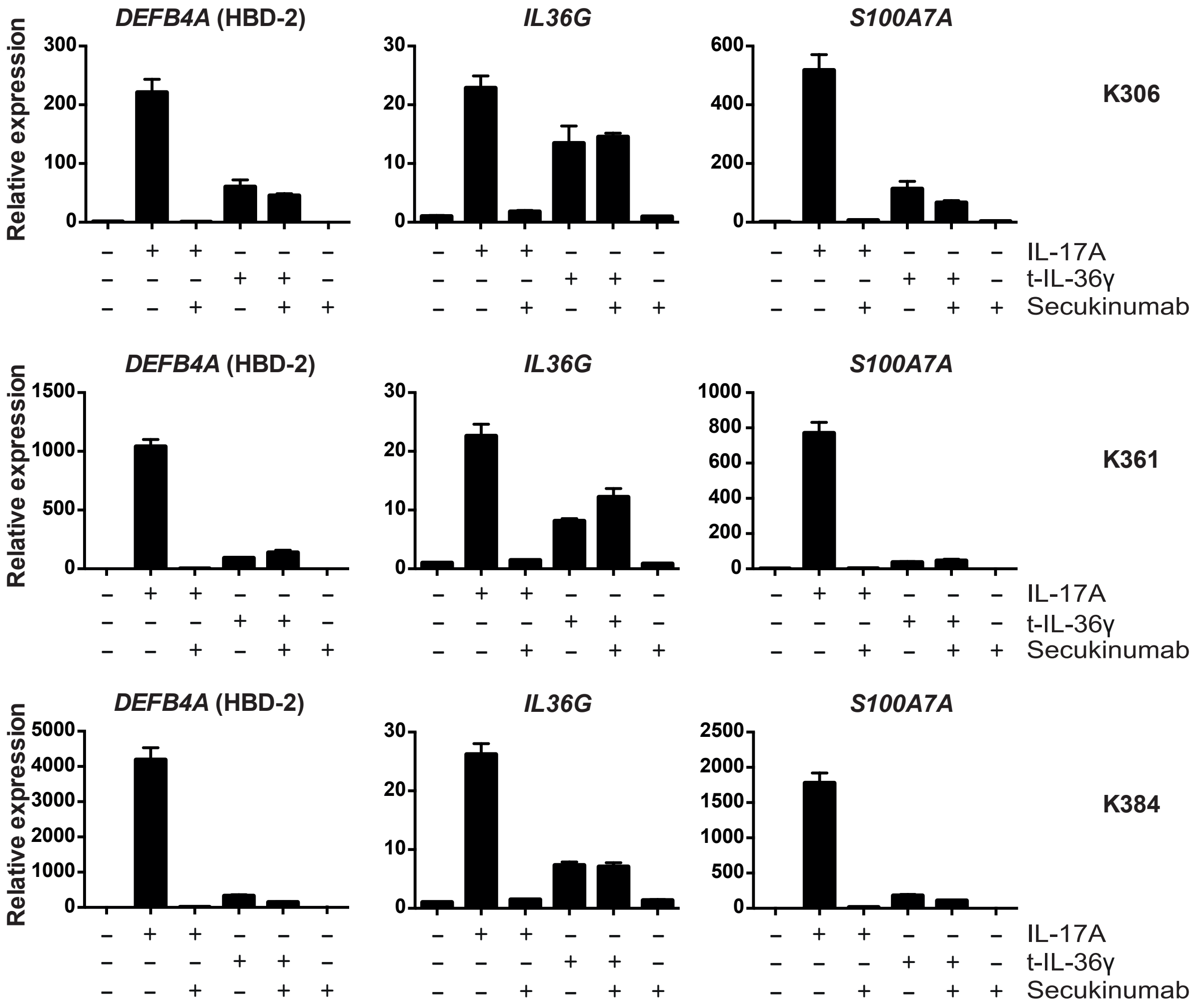

Supplementary Figure S6

Secukinumab interferes with IL-17A but not with IL-36-induced gene expression.

NHEKs stimulated with IL-17A (50 ng/ml) and t-IL36γ (100 ng/ml) and treated with Secukinumab (4.3 µg/ml) as indicated for 48 h. Three individual experiments measured in triplicates are shown. The three experiments were performed with NHEKs of three different donors.

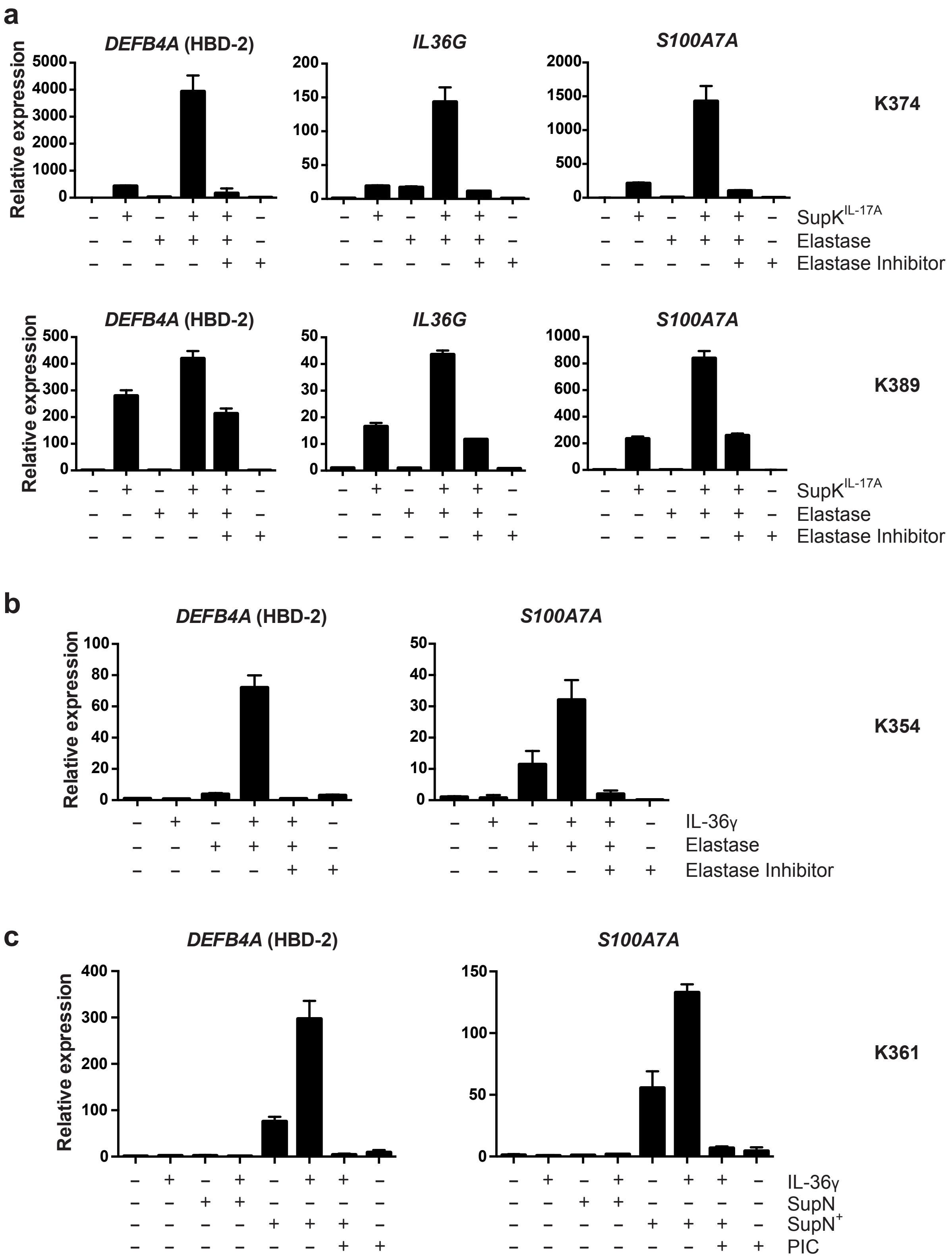

Supplementary Figure S7

Recombinant and IL-17A-stimulated NHEK supernatant-derived IL-36γ is activated by neutrophilic proteases.

(**a**) The supernatant of NHEKs stimulated with IL-17A for 96 h was incubated with elastase (20 nM) and/or elastase inhibitor (200 nM) for 2 h at 37°C. The samples were diluted 1:2 and

subsequently naïve NHEKs were incubated for 16 h. Two individual experiments with technical triplicates are shown.

(**b**) Recombinant elastase (20 nM) was incubated with recombinant IL-36γ (75 ng/ml) and with or without elastase inhibitor for 2 h at 37°C. The samples were diluted 1:2 and naïve NHEKs were stimulated for 16 h. One experiment measured in triplicates is shown.

(**c**) Recombinant human IL-36γ (75 ng/ml) was incubated with the supernatants of neutrophils, which were stimulated with and without PMA, in the presence or absence of protease inhibitor cocktail (PIC) for 2 h at 37°C. The samples were diluted 1:2 and naïve NHEKs were stimulated for 16 h. One experiment measured in triplicates is shown.

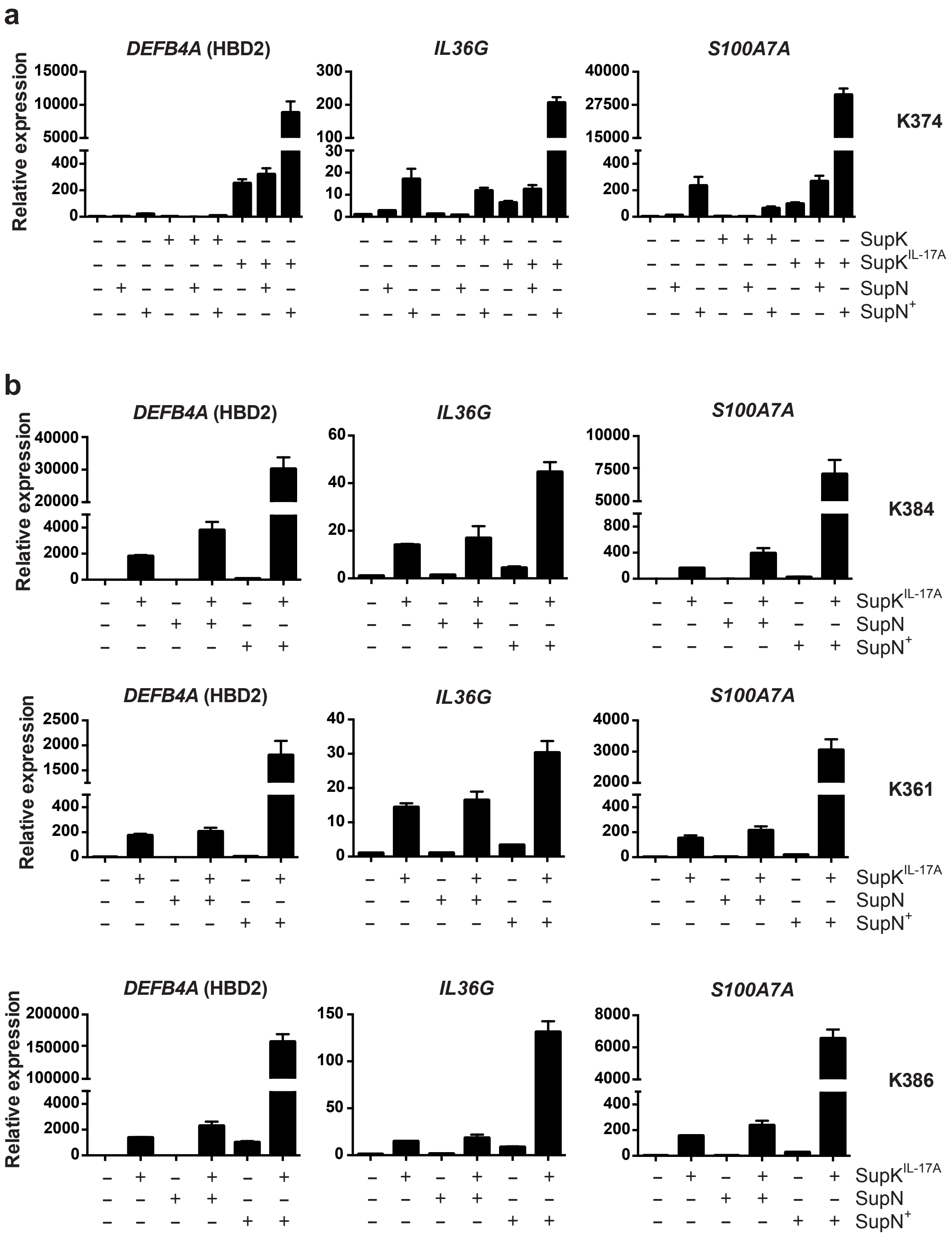

Supplementary Figure S8

The supernatants of IL-17A-stimulated NHEKs and PMA-treated neutrophils cooperate in activating gene expression in naïve NHEKs.

(**a**) The supernatants of NHEKs stimulated with or without IL-17A (50 ng/ml) for 96 h were incubated with the supernatants of neutrophils stimulated with and without PMA for 2 h at

37°C. The samples were diluted 1:2 and naïve NHEKs were stimulated for 16 h. One experiment measured in duplicates with two technical replicates is shown.
(**b**) The supernatants of NHEKs stimulated with IL-17A (50 ng/ml) for 96 h were incubated with the supernatants of neutrophils stimulated with and without PMA for 2 h at 37°C. The samples were diluted 1:2 and naïve NHEKs were stimulated for 24 h. Three individual experiments are shown. The three experiments were performed with NHEKs of three different donors. The mean values ± SD of three technical replicates are shown.

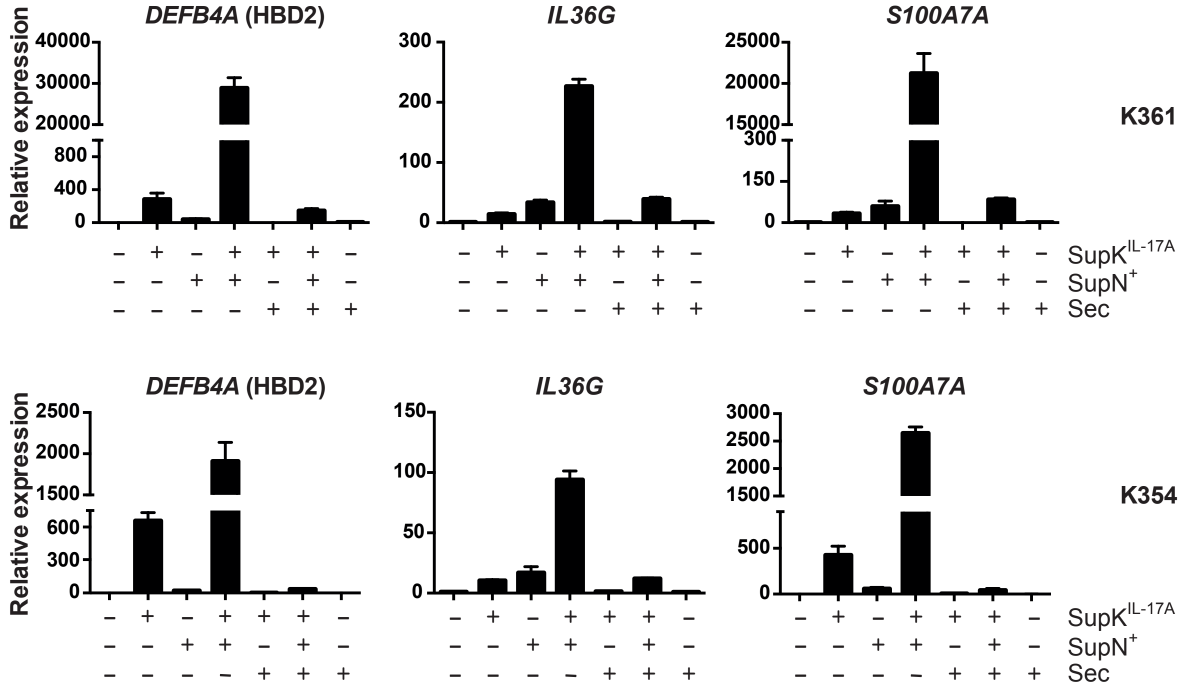

Supplementary Figure S9

The cooperativity of IL-17A-stimulated NHEK supernatants and supernatants of activated neutrophils is inhibited by Secukinumab.

The supernatants of NHEKs stimulated with IL-17A (50 ng/ml) for 96 h were incubated with the supernatants of neutrophils stimulated with and without PMA in the presence or absence of Secukinumab (4.3 µg/ml) for 2 h at 37°C. The samples were diluted 1:2 and naïve NHEKs were stimulated for 16 h. Two biological replicates measured in triplicates are shown.

Belongs to Fig. 1c

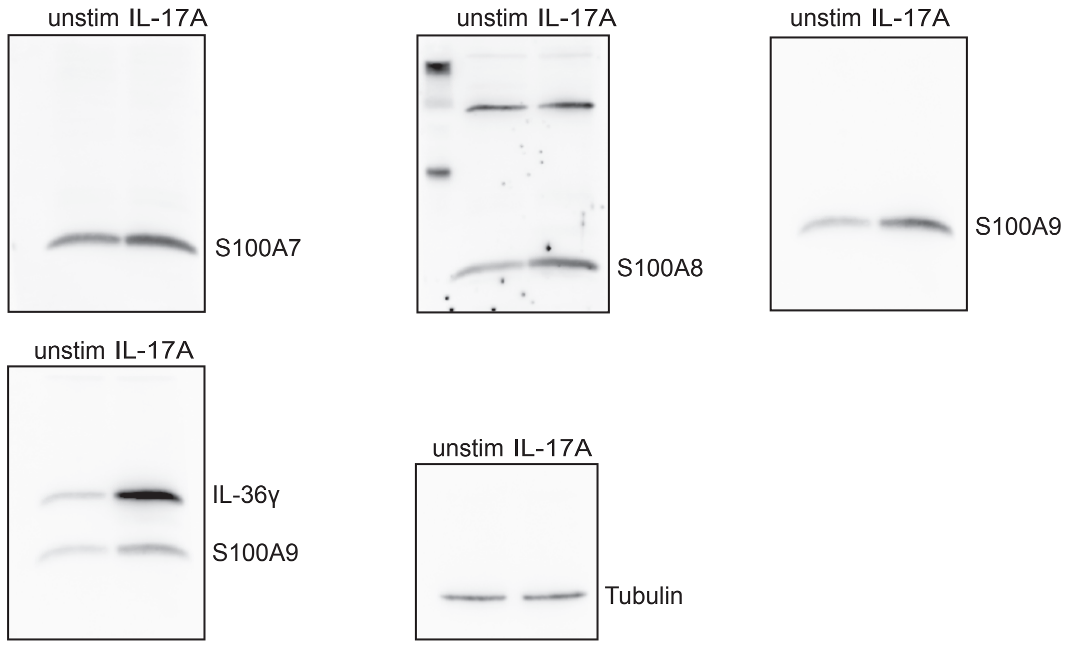

Belongs to Fig. 2b

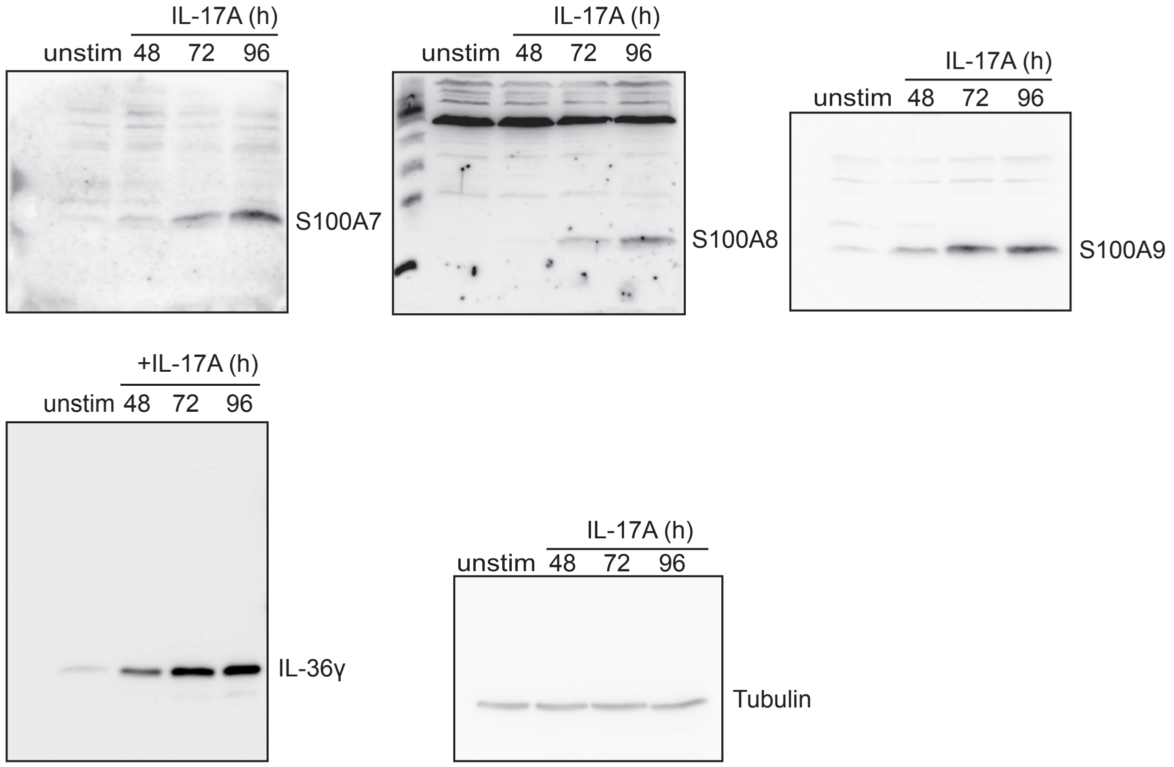

Supplementary Figure S10

Entire Western blots are shown belonging to Figures 1c and 2b.
